# Correction: Bayesian regression explains how human participants handle parameter uncertainty

**DOI:** 10.1371/journal.pcbi.1009932

**Published:** 2022-03-03

**Authors:** Jannes Jegminat, Maya A. Jastrzębowska, Matthew V. Pachai, Michael H. Herzog, Jean-Pascal Pfister

Fig 5 is incorrect. The authors have provided a corrected version here.

**Fig 5:**
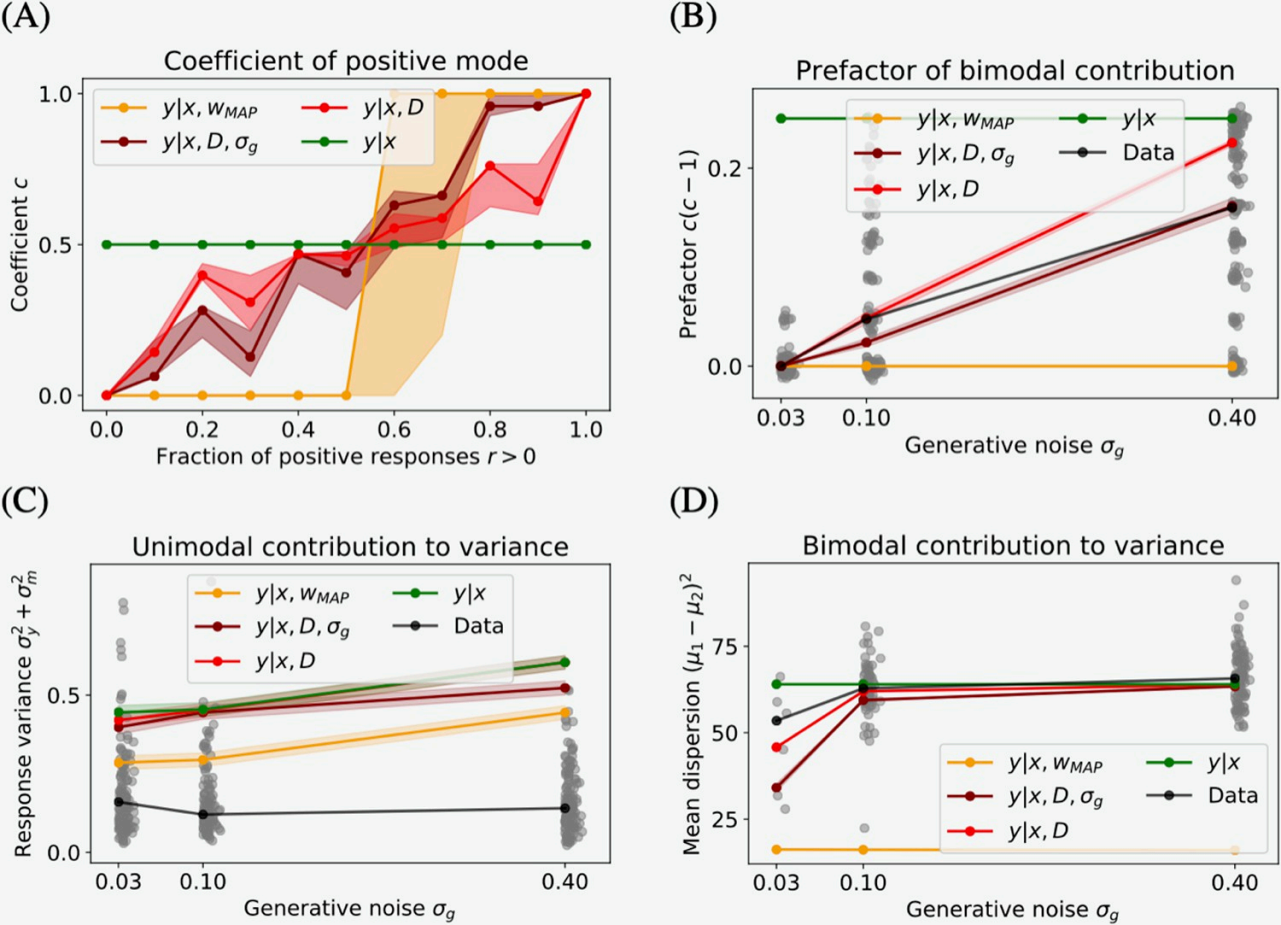
Median of each of the bimodal response distribution variance components across all participants and stimuli. (A) Predicted coefficient of positive mode as a function of the empirical coefficient (across all noise levels). ML-R behaves identically to MAP-R. Thus, the MAP-R curve represents both models. The shaded area shows the 40% and 60% quantiles. (B) Prefactor of bimodal contribution as a function of generative noise. Data jittered for visibility. (C) Unimodal contribution to the variance. Empirical variance computed on mode with majority of responses. (D) Mean dispersion. Only trials with bimodal responses included. As the stimulus becomes more noisy, human responses and B-R variants conform to the prior.
