# Supplementary material for: Bayesian regression explains how human participants handle parameter uncertainty
Source: PLoS Comput Biol. 2020 May 18;16(5):e1007886. doi: 10.1371/journal.pcbi.1007886 (PMC7259793; doi:10.1371/journal.pcbi.1007886)
Supplement: S1 Text — (PDF) [file pcbi.1007886.s001.pdf]

Supplementary Information S1 Text: Bayesian regression explains how human participants handle parameter uncertainty

Jannes Jegminat[1,2*], Maya A. Jastrzębowska[3,4], Matthew V. Pachai[3,5], Michael H. Herzog[3], Jean-Pascal Pfister[1,2]

1 Department of Physiology, University of Bern, 3012 Bern, Switzerland
2 Institute of Neuroinformatics and Neuroscience Center Zurich, ETH and the University of Zurich, 8057 Zurich, Switzerland
3 Laboratory of Psychophysics (LPSY), Brain Mind Institute, School of Life Sciences, École Polytechnique Fédérale de Lausanne (EPFL), 1015 Lausanne, Switzerland
4 Laboratory for Research in Neuroimaging (LREN), Department of Clinical Neuroscience, Lausanne University Hospital (CHUV) and University of Lausanne, 1011 Lausanne, Switzerland
5 Department of Psychology, York University, ON M3J 1P3 North York, Canada

* jannes@ini.uzh.ch

Subject-level analysis of full data set

In the Main Text, we showed the log likelihood averaged across participants. The subject-level analysis in Fig A shows that the average represents a good summary statistics, i.e., it is not dominated by an outlier participant. B-R subject-level for most participants at most experimental conditions.

An interesting difference between the averaged log likelihood and the individual results is the performance of MAP-R at $\sigma_g = 0.03$ Fig A (A). For participants 3, 4 and 7, MAP-R and B-R$_\sigma$ perform almost as well as B-R. Intuitively, this makes sense: as $\sigma_g \to 0$, all models rely fully on the log likelihood and converge to the same prediction. The onset of this convergence effect depends on the participant-specific motor noise σ_m, i.e., when the motor noise becomes the dominant source of noise. For participants 3, 4 and 7, the convergence of the models is more evident than for other participants because the former have higher motor noise.

In the experiments shown in Fig A (B - D), ML-R and MAP-R reflect the averaged behaviour for each subject. B-R clearly outperforms the other models. P-R and B-R$_\sigma$ perform worse than B-R for most participants and conditions but win occasionally, i.e., for participant 1 in (B) and participants 3, 4 and 6 in (C). However, the margin by which P-R or B-R$_\sigma$ outperform in these cases is small, and the general ordering of the models is represented well by the averaged performance. In conclusion, the average across participants provides an informative summary of the data.

Subject-level analysis of unimodal data set

Here, we report the log likelihood analysis of the unimodal data set for each participant. The subject-level results confirm the group level results reported in the Main Text: B-R with sampling generally performs equally well or better than the other models.

The left column in Fig B shows the performance of sampling-based models and the right column – that of the loss function models. B-R with sampling is used as the baseline in both cases. In the first and last conditions ((A, B) and (G, H), respectively), the average across the participants represents the individual results well. Participants 4 and 7 are the only exceptions. In these conditions, B-R with sampling outperforms the other models strongly.

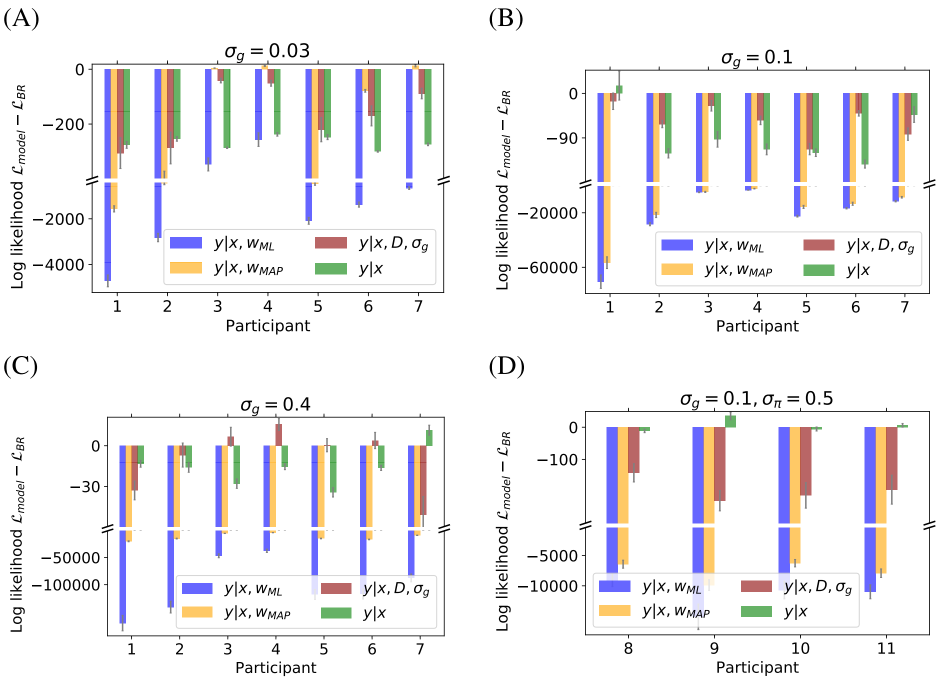

Fig A. Subject-level model comparison. Same data as that shown in Fig 3 in the Main Text. One set of participants (1-7) participated in the main experiment ((A)-(C)) and a different set of participants (8-11) participated in the experiment reported in (D). With some exceptions, the average across participants (reported in the Main Text) represents the individual performance well. Error bars indicate SEM computed across unique stimuli.

At $\sigma_g = 0.1$ (C, D), most models, both sampling and loss-based perform within a close margin of each other. Only in the case of participant 1 do MAP-R, P-R and the loss-based models clearly perform worse than the B-R variants. At $\sigma_g = 0.4$ (E, F), a similar pattern emerges. For most participants, the differences in performance between the models, sampling and loss-based alike, are small. Given that the stimulus has a high noise level, this is to be expected to some degree. Only in the case of participant 1 does B-R with sampling clearly outperform the other models. Meanwhile, in the case of participant 2, the loss-based models perform much worse than sampling models. Moreover, the performance plot does not provide strong evidence in favour or against any of the models. In summary, the average across participants only reflects the behaviour of the majority of the participants. Most exceptions can be found in the performance of participants 3, 4 and 7. At $\sigma_g = 0.1$, the average is dominated by a single subject. Despite a higher degree of inter-subjective variability compared to the bimodal data set, the presented analysis either favours B-R with sampling or provides no strong evidence favouring any one of the models in particular.

No evidence for substantial learning

We investigated whether participants learn over the course of the experiment. One potential explanation for learning is that subjects become better at the task over time, i.e., their internal model conforms more and more closely to the generative model. Another explanation is that participants improve their performance by repetition, i.e., they memorize the generative parabola associated to a stimulus (rather than solving the regression problem). To understand better if these factors played a role, we analysed learning with two different metrics.

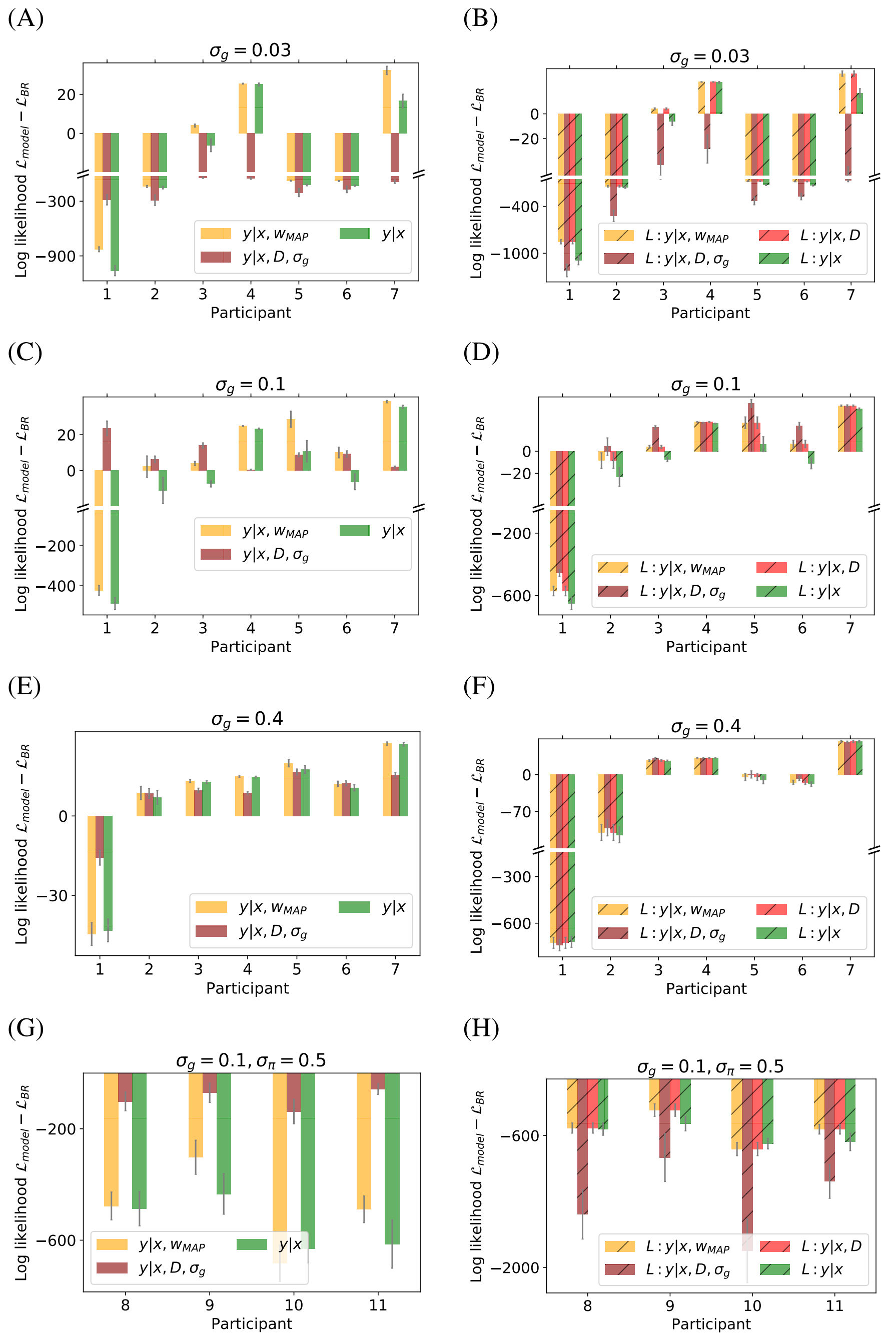

Fig B. Unimodal subject-level model comparison. Same data as that shown in Fig 6 in the Main Text. Each row represents an experimental condition. (A, C, E, G) Performance based on samples from the predictive distributions. (B, D, F, H) Performance of the loss function version of the same models. The presentation is analogous to the presentation in the Main Text. Error bars indicate the SEM computed across unique stimuli.

The distance between responses and the generative parabola remains constant

For both metrics, we split trials into two halves according to the number of times the same stimulus had been repeated. For a stimulus repeated 20 times in total, this means that the 10th

repetition belongs to the first half, even if it occurred late in the experiment. Fig C shows the distance between the generative parabola and the participants' responses for all four experimental conditions. Each marker corresponds to a participant. Markers below the dashed line indicate learning, i.e., smaller distances to the generative parabola in the second half of repetitions. We used Bayesian regression to fit a linear model across conditions Fig C (A) and per condition Fig C (B-E), see Eq (S3). The diagonal line (dashed black that would indicate an absence of learning lies within the confidence intervals of the model fit (green). Thus, this metric provides evidence against the presence of learning.

Jumps between modes occur throughout the entire experiment

Since the idea that participants sample from a bimodal distribution is central to this work, we made the following adaptions to the first metric: for each unique (frozen noise) stimulus, we counted the number of times that participants' respsonses oscillated between the upper and lower mode in the first and second half of presentations. Fig D shows the number of jumps in the first and second half of repetitions. In the absence of learning, the data should lie on the dashed line with slope one. We use the same procedure as before to obtain a model with confidence intervals. In the first condition Fig C (B), the data is centered closely around the origin, i.e. very few jumps occur. However, this concentration around a single point limits the usefulness of a linear fit. In the condition $\sigma_g = 0.1$ (C), the linear fit (green) reveals that less jumps occurs in the second half of the repetitions but jumps remain present throughout the second half. This suggests a mild form of learning under this condition. In the other two conditions (D, E), the data is compatible with the assumption that no learning occurs. This metric suggests that a weak form of learning takes place during the condition $\sigma_g = 0.1$ but not during the other conditions. In particular, subjects flip between the modes even at later repetitions of the same stimulus. This implies that the bimodal response distributions do not result from a one time switch between the modes (e.g., as a result of learning of the generative parabola) but occur throughout the entire experiment.

Log likelihood performance remains constant

Finally, for qualitative inspection, we report the log likelihood averaged over participants as a function of repetitions and time bins. The baseline model is Bayesian regression and all models use sampling as the decision model. For the sake of comparability, we matched the number of time bins to the number of repetitions by averaging trials associated with one bin. Fig E shows that performance is constant for most models and conditions. Comparing the left and right columns to each other, we find no evidence that performance over trials differs from performance over repetitions. This was expected since the two (repetition number and trial number) are correlated, e.g. the 20th repetition of a unique stimulus occurs rather late in the experiment.

Neither of the two metrics nor the performance time series offers substantial proof of learning. Fig D (B-D) in particular shows that the bimodality of responses is present throughout the course of the experiment. This justifies the assumption used in the analysis in the Main Text that model performance can be evaluated independently of time.

Linear Bayesian regression fit to learning data

To fit the learning data $D = \{\tilde{x}_i, y_i\}_{i=1}^N$ in Fig D and Fig C, we used linear Bayesian regression. We assumed a Gaussian likelihood:

$$y = \theta_1 \tilde{x} + \theta_0 + \sigma\eta = \theta^T x + \sigma\eta, \tag{S1}$$

where $\eta \sim \mathcal{N}(0, 1)$, σ is a free parameter and $x = (1, \tilde{x})$. As a prior for the parameter, we used a Gaussian with mean $\mu_0 = (0, 1)$ and (initially) diagonal precision matrix $\mathbb{I}$. Then, the posterior

over θ has precision and mean:

$$A = \mathbb{I} + \sigma^{-2} \sum_i x_i x_i^T$$
$$\mu = A^{-1} \left(\mu_0 + \frac{\sum_i x_i y_i}{\sum_i x_i x_i^T} \right). \quad \text{(S2)}$$

The posterior predictive $p(y|x, \mathcal{D}, \sigma^2) = \mathcal{N}(y; \mu_y(x), \sigma_y^2(x))$ has mean and variance:

$$\sigma_y^2 = \gamma \sigma^2$$
$$\mu_y = \gamma \mu^T A (A + \sigma^{-2} x x^T)^{-1} x, \quad \text{(S3)}$$

where $\gamma = (1 - x^T(\sigma^2 A + x x^T)^{-1} x)^{-1}$. While the hyperparameters of the prior do not affect the fit much, the noise level σ^2 is crucial for determining the confidence intervals of the fit. Thus, we determined σ^2 numerically as the maximizier of the model evidence.

Experiment

Participants

Seven naive participants (3 females, 4 males, ages 21-27) participated in the main experiment and four naive participants (all males, ages 21-30) took part in the second experiment. All participants had normal or corrected-to-normal visual acuity, as measured with the Freiburg Visual Acuity Test (Bach, 1996). Participants were paid 20 CHF/hour. All participants gave informed consent in accordance with protocol 384/2011 “Commission cantonale d’éthique de la recherche sur l’être humain”. Participants provided written consent prior to the experiment.

Apparatus and Stimuli

The experiments were programmed using custom software implemented in MATLAB with the Psychophysics Toolbox (Brainard, 1997). Stimuli were presented on a gamma-corrected ASUS VG248QE LCD monitor with a resolution of 1920x1080 (36 pixels/cm) and refresh rate of 120 Hz. Stimuli consisted of four black points (5-arcmin diameter) sampled from the generating parabola described and jittered vertically with Gaussian noise of standard deviation. On each trial, the width of the generating parabola was sampled from the prior distribution (as described in the methods section of the main text).

Participants viewed the display binocularly, and a chin rest stabilized viewing distance at 75 cm from the screen. The origin of the Cartesian coordinate system (50 pixels/unit), according to which the positions of the stimulus points were defined, was placed at the center of the screen. Each trial comprised a fixation dot (5 arcmin diameter) presented at the center of the screen for 1 s followed immediately by presentation of the stimulus. Along with the four stimulus points, a red point of the same size was presented 2 units to the right of the screen center and participants were instructed to adjust this point up or down using the arrow keys to indicate the y-axis location at which the generating parabola would pass. Participants were given unlimited time to make their response.

In order to allow for learning of the prior distribution of the quadratic parameter, feedback was provided after each trial by overlaying on the stimulus points the generating parabola with a green line following each response. A 1 s delay (with fixation) preceded the next trial.

Computation of predictive distributions

In order to compute the predictive distributions of B-R, B-R$_\sigma$ and P-R, we need two sets of update equations for Gaussians found in any standard text book on statistics (e.g. Bishop (2006)).

For convenience, we call them inference and extrapolation. Inference represents an update of a Gaussian prior $p(w|\mu, \sigma^2)$ with the likelihood $p(\{y_i\}_{i=1}^4 | \{x_i\}_{i=1}^4, w, \sigma_g^2)$. The result is the posterior, here indicated by the subscript ρ:

$$\sigma^{-2} \to \sigma_\rho^{-2} = \sigma^{-2} + \sigma_g^{-2} \sum_{i=1}^{4} x_i^4$$
$$\mu \to \mu_\rho = \frac{\sigma_\rho^2}{\sigma^2}\mu + \frac{\sigma_\rho^2}{\sigma_g^2} \sum_{i=1}^{4} x_i^2 y_i$$
$$c \to c_\rho = c\frac{\sigma_\rho}{\sigma} \exp\left(-\tfrac{\mu^2}{2\sigma^2} + \tfrac{\mu_\rho^2}{2\sigma_\rho^2}\right), \tag{S4}$$

where the last equations represents the (unnormalised) change of a mixture coefficient.

The extrapolation equations corresponds to transforming a Gaussian distribution $p(w|\mu, \sigma^2)$ into the predictive distribution at $x_\star$ via the likelihood term $p(y|x_\star, w, \sigma_g^2)$. This step corresponds to the marginalisation over the parameter w. We indicate the predictive by the subscript y:

$$\mu_y = \mu x_\star^2$$
$$\sigma_y^2 = \sigma_g^2 \left(1 + \frac{\sigma^2 x_\star^4}{\sigma_g^2}\right). \tag{S5}$$

For P-R, we apply Eq (S5) to both components of the prior. For B-R, we apply Eq (S4) to both components of the prior. Then we use Eq (S5) on both components. Finally, we normalise the sum of coefficients of the resulting mixture distribution to unity. B-R$_\sigma$ differs from B-R only in that the true noise parameter is replaced by the following maximum likelihood approximation:

$$\hat{\sigma}_g^2 = \frac{1}{4} \sum_{i=1}^{4} (y_i - w_{\mathrm{ML}} x_i^2)^2 \text{ with } w_{\mathrm{ML}} = \frac{\sum_{i=1}^4 y_i x_i^2}{\sum_{i=1}^4 x_i^4}. \tag{S6}$$

The predictive distribution of ML-R is a Gaussian with mean $w_{\mathrm{ML}} x_\star^2$ and variance σ_g^2. The predictive distribution of MAP-R uses $w_{\mathrm{MAP}} = \arg\max_w p(w|D)$ instead of w_{ML}, i.e., the parameter that maximises the posterior. We obtain this value numerically.

Noisy inference vs Bayesian regression

Here we want to study to which extent a model based on noisy inference could explain our results. Drugowitsch et al. (2016) showed that human response variability in a categorical decision task can be attributed to noisy inference rather than noisy decision making or perceptual uncertainty. In their framework, each type of noise is modelled as a Gaussian with noise level $\sigma_{sen}, \sigma_{\mathrm{inf}}$ and σ_{sel} for sensory, inference and selection noise.

We assume that sensory noise σ_{sen} plays no role in our task because the 4-dot stimulus is clearly visible and shown until participants make their decision. Drugowitsch et al. propose that the inference noise σ_{inf} is added to the log likelihood of each category before applying a sigmoidal decision function to the difference of log likelihoods.

In the context of our task, the categories correspond to the upwards or downwards parabola. The log likelihoods are:

$$\mathcal{L}_\pm = -\tfrac{1}{2\sigma_g^2} \sum_{i=1}^{4} (y_i - \mu_\pm x_i^2), \tag{S7}$$

with the prior mean $\mu_\pm = \pm 1$. The difference between both log likelihood is:

$$\Delta\mathcal{L} = \tfrac{1}{\sigma_g^2} \sum_{i=1}^{4} y_i x_i^2 (\mu_+ - \mu_-) = 2\frac{w_{\mathrm{ML}}\mu_+}{\sigma_g^2} \sum_{i=1}^{4} x_i^4, \tag{S8}$$

where we substituted Eq (S6) in the last step. Because of the symmetrical prior, the sign of the Maximum likelihood estimator determines the sign of the log likelihood difference.

Following Drugowitsch et al. (see their Supplementary Information, Section 2.7), we first use a cumulative Gaussian as sigmoidal non-linearity for decision making but later replace it by logistic sigmoidal. The advantage is that the Gaussian expectation over the noise is easily computed for a cumulative Gaussian Φ but the logistic sigmoidal can be expressed in terms of its arguments more easily. We encode the decision noise in the sigmoidal function and add the Gaussian inference noise to both log likelihoods (resulting in a factor $\sqrt{2}$ in the standard deviation. The probability of selecting the positive parabola is:

$$\begin{aligned}\hat{c}_+ := P(+|\Delta\mathcal{L}) &= \left\langle \Phi\left(\frac{\Delta\mathcal{L} + \sqrt{2}\sigma_{\mathrm{inf}}\eta}{\sigma_{\mathrm{sel}}}\right)\right\rangle_{\eta\sim\mathcal{N}(0,1)} \\ &= \Phi\left(\frac{\Delta\mathcal{L}}{(\sigma_{\mathrm{sel}}^2 + 2\sigma_{\mathrm{inf}}^2)^{1/2}}\right) \approx \frac{1}{1+\exp(-\beta\Delta\mathcal{L})},\end{aligned} \tag{S9}$$

where the contributions of both noises and the approximation factor between cumulative Gaussian and logistic sigmoidal have been summarized in $\beta = \frac{\pi}{\sqrt{6}\sqrt{\sigma_{\mathrm{sel}}^2+2\sigma_{\mathrm{inf}}^2}}$. Assuming loss-based decision making, the posterior predictive distribution of noisy inference is:

$$p(r|x, \mathcal{D}_j, \beta) = \hat{c}_+\mathcal{N}(r; \mu_+ x_\star^2, \sigma_m^2) + (1-\hat{c}_+)\mathcal{N}(r; \mu_- x_\star^2, \sigma_m^2), \tag{S10}$$

where $x_\star$ is the x-location of the line on which the responses is requested and σ_m^2 represents the contribution of the motor noise.

Next, we express the mixture coefficient c_+ of the positive mode in Bayesian regression in an analogous form. Recall that the posterior and posterior predictive share the same mixture coefficient in our experiment. Thus, it suffices to consider the mixture coefficient of the posterior. The unnormalised mixture coefficient $c_\rho^\pm$ for both modes was computed in Eq (S4). By normalization we have:

$$c_+ = \frac{1}{1 + c_\rho^-/c_\rho^+} = \frac{1}{1+\exp(-\frac{\sigma_\rho^2}{\sigma^2}\Delta\mathcal{L})}, \tag{S11}$$

where we used that both modes of the prior (as well as the posterior) are the same and that the prior means satisfy $\mu_+ = -\mu_-$. While the prefactor β in the noisy inference algorithm (see Eq (S9)) is a model parameter, the prefactor $\frac{\sigma_\rho^2}{\sigma^2}$ in Bayesian regression depends weakly on the current stimulus:

$$\frac{\sigma_\rho^2}{\sigma^2} = \frac{1}{1 + \frac{\sigma^2}{\sigma_g^2}\sum_{i=1}^4 x_i^4}. \tag{S12}$$

The predictive distributions of noisy inference regression and Bayesian regression are strikingly similar. In particular, both models predict a transition from unimodal to bimodal responses as σ_g increases. An important difference between the models is that noisy inference relies on fitting the parameter β to the participants' responses. The parameter controls how sensitively the transition depends on σ_g. In Bayesian regression, a data dependent prefactor takes the role of the prefactor.

A minor difference is the mean and variance of the modes of the predicted response distribution differ. Bayesian regression uses the posterior mean and variance to make predictions. In contrast, noisy inference (as shown here) relies on the prior means and the motor noise determines the variance of the modes.

In summary, despite the similarities between the B-R model and the noisy inference model, the main difference is that the noisy inference model relies on a free parameter (β) that needs to be fitted. In order to keep the whole analysis fitting free, we did not include this model in the model comparison.

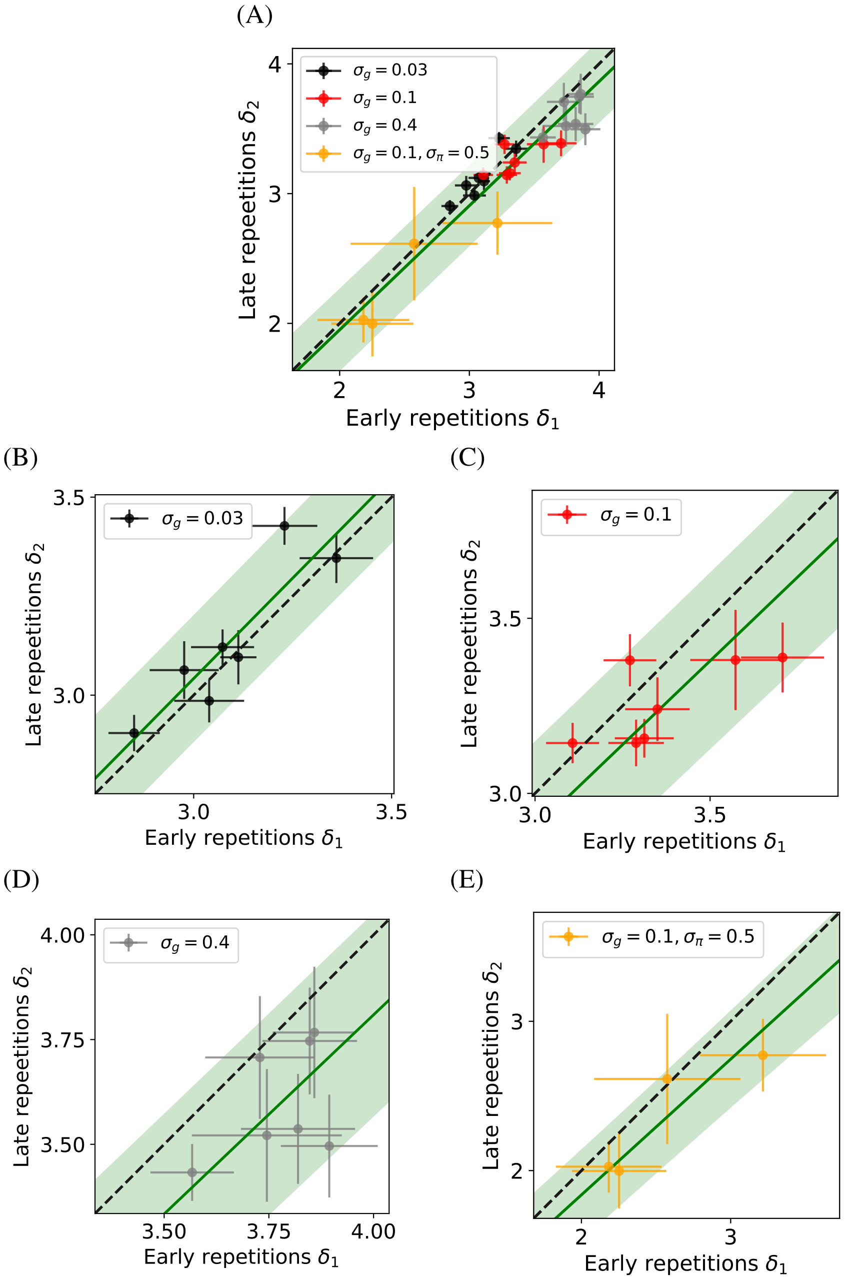

Fig C. Stability of responses in early and late repetitions in terms of distance to the generative parabola. No substantial learning is observed in the four noise conditions. The x-axis shows the distance between response and intersection of the $x^\star = 2$ line with the generative parabola $\delta_1 = \frac{2}{n}\langle\sum_{i=1}^{n/2}(w_j x_\star^2 - r_i^{(j)})\rangle_j$ computed on the first half of repetitions of repeated stimuli and averaged. The y-axis shows the analogous quantity computed on the second half of repetitions: $\delta_2 = \frac{2}{n}\langle\sum_{i>n/2}^{n}(w_j x_\star^2 - r_i^{(j)})\rangle_j$. Dots below the dashed line indicate learning. The fit (green) is a linear Bayesian regression model with 2-SD as confidence intervals. (A) Fit to data from all participants and all conditions. (B-E) Individual fit per noise condition. The no-learning (diagonal black dashed line) condition falls within the confidence intervals (green). Error bars represent the SEM computed across unique stimuli. (E) The orange error bars are larger because responses were more variable and the number of repetitions is lower.

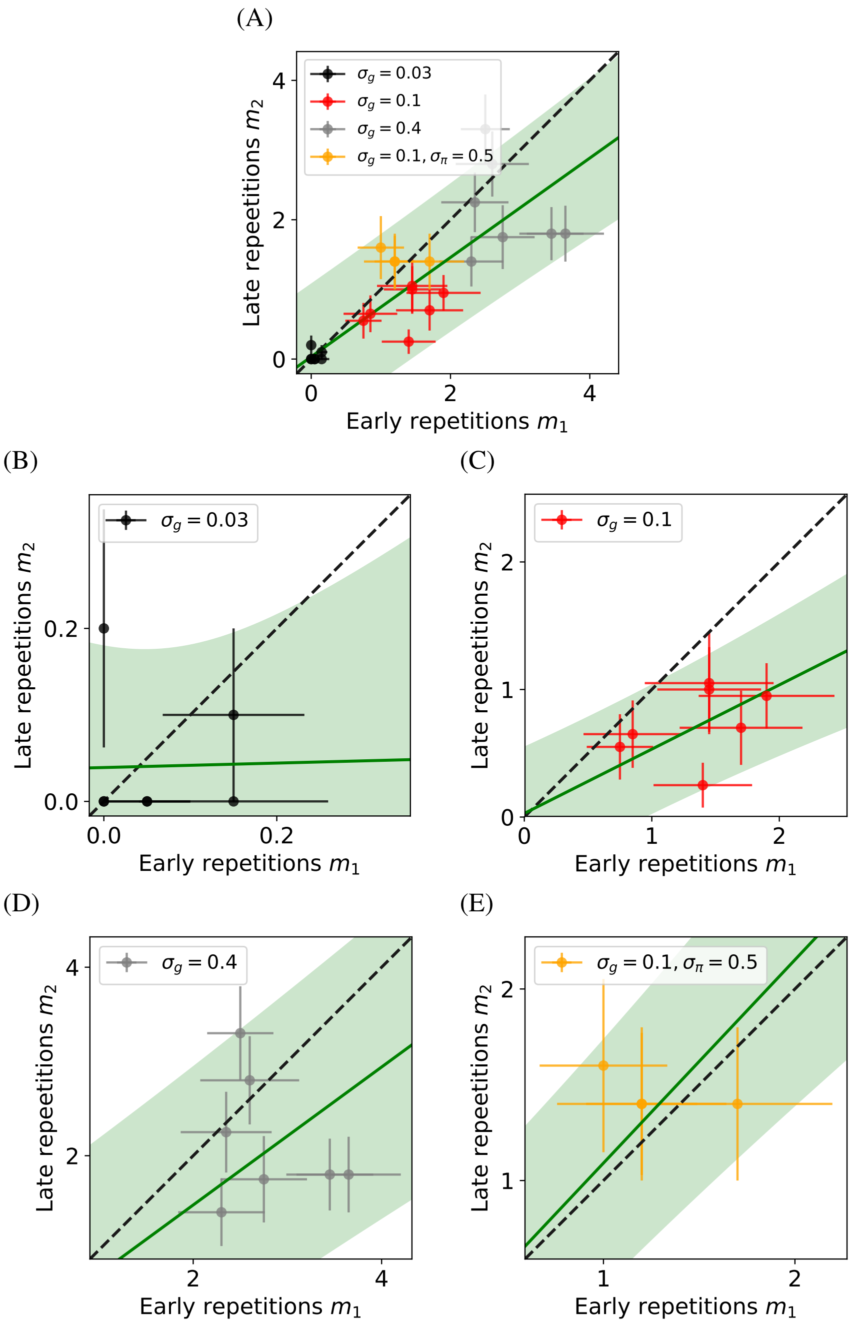

Fig D. Stability of responses in early and late repetitions in terms of mode switching. No substantial learning is observed in the four noise conditions. The x-axis shows the average number sign changes of a participant's response between successive presentations of a repeated stimulus $m_1 = \langle \sum_{i=1}^{n/2-1} (1 + \mathrm{sign}(\mathrm{r}_\mathrm{i}^{(\mathrm{j})} \mathrm{r}_{\mathrm{i+1}}^{(\mathrm{j})}))/2 \rangle_\mathrm{j}$. The y-axis shows the analogous quantity computed on the second half of repetitions: $m_2 = \langle \sum_{i>n/2}^{n/2-1} (1 + sign(r_i^{(j)} r_{i+1}^{(j)}))/2 \rangle_j$. Intuitively, m counts the number of jumps between the positive and negative mode. Dots below the dashed line indicate learning. (A) Fit to data from all participants and all conditions. (B) The data is centered around the origin (as expected at low noise) such that the linear fit is unreliable. (C) A reduction in jumps between modes suggests a mild form of learning under the $\sigma_g = 0.1$ condition. (D, E) The number of jumps between the modes does not change substantially between the first and second halves of repeated stimuli.

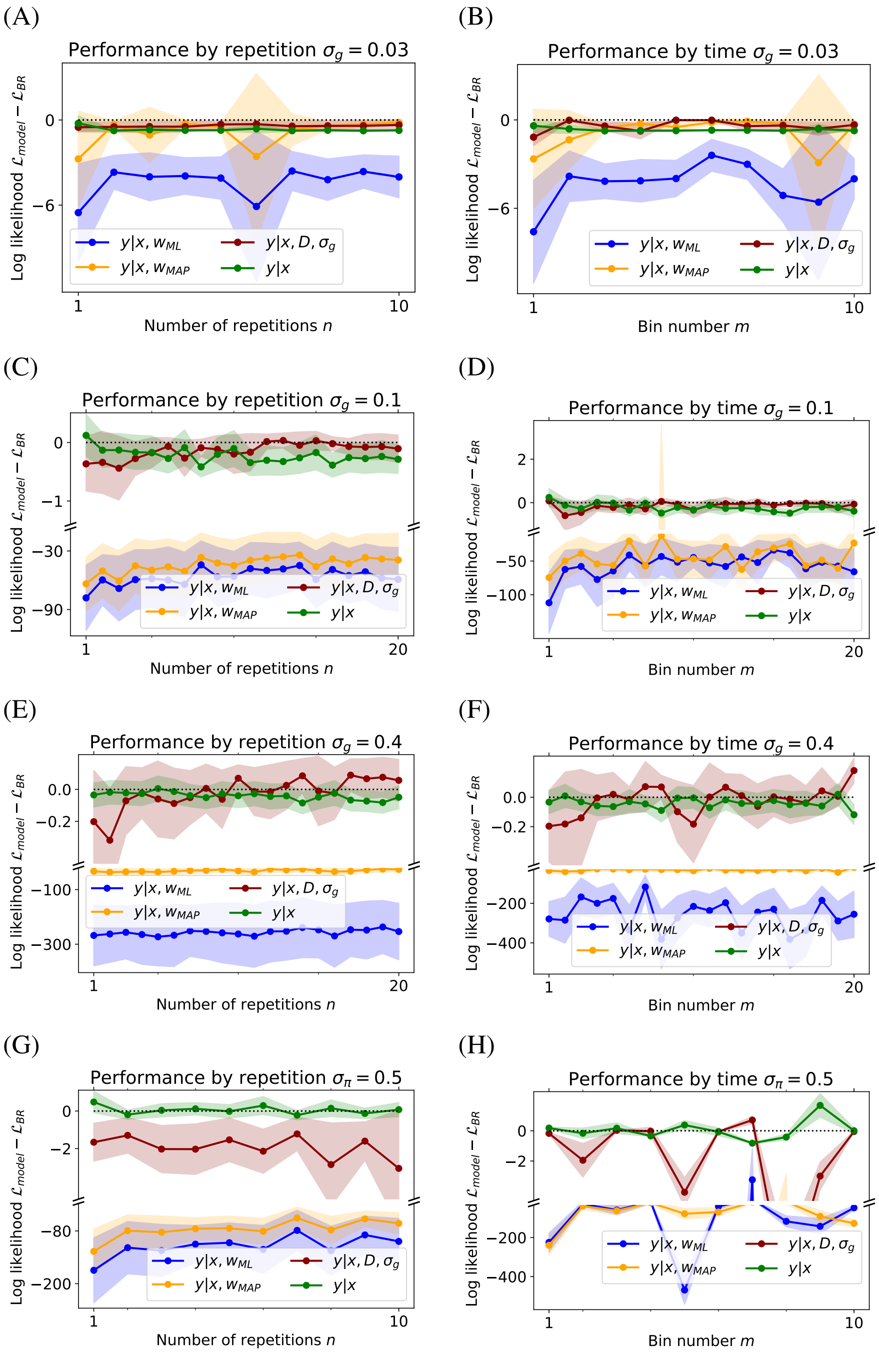

Fig E. Performance over time. Performance, as quantified by the log likelihood with respect to B-R, averaged across participants, is stable over trials and repetitions. Each row corresponds to an experimental condition. (A, C, E, G) Performance averaged across stimuli as a function of repetition. $\sigma_g = 0.03$ has only 10 repetitions because the experiment was split up into two sessions. (B, D, F, H) Performance as a function of time bin is stable. Number of bins matched to the number of repetitions on the right. The shaded area represents the SEM across participants.
