# Supplementary material for: Bayesian regression explains how human participants handle parameter uncertainty
Source: PLoS Comput Biol. 2020 May 18;16(5):e1007886. doi: 10.1371/journal.pcbi.1007886 (PMC7259793; doi:10.1371/journal.pcbi.1007886)
Supplement: Attachment — Submitted filename: Response to Reviewers Revision2.pdf [file pcbi.1007886.s004.pdf]

Reviewer's Responses to Questions

Comments to the Authors:
Please note here if the review is uploaded as an attachment.

Reviewer #1: I appreciate the authors' changes to the manuscript. I think that the model comparison, as well as the analysis of the response variance is much improved. I don't have any major comments on the details of the analysis and its description. Two issues nonetheless remain, as I will describe in turn.

The first is that not all statements in the manuscript are immediately supported by the performed analyses. This is a thread that goes through the whole manuscript, such that pointing out every single instance would be too tedious. Some of the most glaring instances are:

* Probability matching/sampling: the manuscript states that human participants perform sampling/probability matching, but the authors haven't tested alternative models of noisy inference. The latter requiring to fit parameters might be a reason for the authors to not want to test them, but it is not a reason for ruling out the possibility that the observed variability is due to noisy inference. Thus, all statements that state that humans choose by probability matching/sampling needs to be toned down/relativized. This starts with the abstract ("We further add evidence that humans use probability matching rather than Bayesian decision theory..." - a noisy inference model might explain the data equally well), over the Results (pages 6/7), to the Discussion. The discussion at least mentions the possibility of noisy inference as an open question, but the rest of the manuscript should word its conclusions more carefully - in particular in the light of increasing evidence (e.g., from Wyart lab) that behavioral variability that has been previously ascribed to posterior sampling can be equally well (or better) explained by noisy inference. Thus, strongly stating that participants sampled might, in fact, be wrong.
We thank the Reviewer for pointing out that our conclusion about sampling was too strong, given that we did not include noisy inference in the model comparison. To make this limitation clearer, we introduced the distinction between *exact inference* and *noisy inference*; and explicitly constrain the scope of the study to exact inference.

On p3, in the preview of the results, we added the words "exact inference" to make this scope clear:
"Indeed, a loss based model with exact inference cannot explain the bimodality of the participants' response distributions"

On p5, we explain the distinction between exact and noisy inference:
"For all models considered here the predictive distribution depends deterministically on the 4-dot stimulus. In this sense, they rely on exact inference. Noisy inference is an alternative which assumes that the inference process is corrupted by noise \cite{drugowitsch2016computational}. This alternative would require an additional noise parameter which governs the level of inference noise (see Supplementary Information). Here, we constrain ourselves to models with exact inference to remain fitting-free […]."

In the caption of Fig 3, we added the words "exact inference" to clarify that our conclusions apply only to exact inference models:

“At all other conditions and for all regression models, sampling explains the data better than loss-based decision models with exact inference.”

On p7, we added the words “exact inference” to the conclusions drawn from the model comparison. The sentence now reads:
“The second explanation for the better performance of sampling is that the loss function approach with exact inference underestimates response variability.”

Throughout the manuscript, we remind the reader that our conclusions are conditioned on the “models considered here”, e.g. on p11 at the end of the unimodal analysis:
“[…] the unimodal analysis provides convincing evidence of the superiority of B-R with sampling over other models considered here.”

On p12, in the discussion of our results for sampling, we soften the conclusion by replacing the words “provide evidence” by “suggest”:
“Our results suggest that humans turn the posterior predictive distribution into a response via probability matching rather than Bayesian decision theory.”

* Abstract: "Our results show that humans use Bayesian regression" - they suggest, but don't show
We agree with the Reviewer that our initial formulation was too strong. We added the phrase “under our specific experimental conditions” to make the scope of the conclusion clear. We also updated the last sentence of the abstract by replacing the words “we suggest” with “our results support the hypothesis”.
The modified abstract reads: “Our results show that, under our specific experimental conditions, humans behave in a way that is consistent with Bayesian regression. Moreover, our results support the hypothesis that humans generate responses in a manner consistent with probability matching rather than Bayesian decision theory.”

* p8: "An explanation for this discrepancy is that participants estimated the noise on a trial-by-trial basis" - this sounds as if you _knew_ that participants performed this estimation. Wouldn't it be better to call this "A potential explanation"?
We thank the Reviewer for the suggestion and added the word “potential” in front of “explanation”.

The second is an overall lack of precision in parts of the manuscript. There are multiple ambiguous/imprecise statements, and leaving it up to the reader to deal with them might lead to misunderstandings. Thus, I urge the authors to resolve any potential ambiguities in their writing. Examples include:
We thank the Reviewer for this constructive comment and for the meticulous read of the manuscript. To address this point, we replaced ambiguous words in the manuscript with more precise formulations. Because we modified several words or sentences in each paragraph, we do not give an exhaustive list here but refer to the marked-up version of the manuscript. However, we exemplify the type of modifications with three examples:

In the caption of Fig 3 a pair of sentences read:
“For B-R, B-R$_\sigma$ and P-R, loss-based models do not predict bimodal responses. At low noise $\sigma_g = 0.03$, they underestimate the response variance.” In the new version, “they” was replaced by a repetition “loss-based models”.

Caption of Fig 5, we replaced “Median of the distribution over […]” by the more precise formulation “Median of each of the bimodal response distribution variance components across all […]”

On p11, we replaced “[…] MAP-R captures some participants well […]” by the more precise formulation “[…] MAP-R reflects the responses of some participants well […]”.

* p3: "The proposal is to apply Bayesian regression to DNNs." - who proposes this? Are you? Do others?
We agree with the Reviewer that we did not make clear that “the proposal” is the proposal of the entire literature of BNNs. This might be confusing in particular if one is not aware of the fact that Bayesian regression applied to DNNs defines BNNs. To make this clearer, we placed the sentence right after the technical introduction of BNNs in terms of weight uncertainty: “Thus, the proposal of BNNs is simply to apply Bayesian regression to DNNs. BNNs promise better performance in the low data regime.”

* p3: "The presentation order was randomized" - this is imprecise. It was randomized within each noise level, but blocked across noise levels.
We changed the sentence to:
“The stimulus presentation order was randomized within each noise level.”

* p3: "The advantage of a response distribution over single responses" - this is contradictory, as a single response doesn't give a distribution. Should this be "The advantage of observing several responses for the same exact stimulus is that we can compare the set of responses to the response distribution predicted by the different models"?
We changed the sentence to:
“The advantage of observing several responses for the same exact stimulus is that we can compare the observed response distribution to the response distributions predicted by the different models in terms of variance and bimodality.”

* p5: "[...] motor noise is the only source of uncertainty" - do you mean that it is the only source of response variability? Uncertainty is never measured.
We replaced “uncertainty” by “response variability”.

* p7: "we use the model likelihood p(M|Rk)" - p(M|Rk) is the model posterior, not the model likelihood, which would be p(Rk|M).
We agree with the Reviewer that we should have referred to “model posterior” and updated the sentence. We also changed the notation from R_k to R^{(k)} to be consistent with R_j, the set of responses to a stimulus D_j. We added the precise definition of R^{(k)} on p4.

* p7: "shows that the majority of the population" - do you mean that the majority of the participants' responses?
We thank the Reviewer for the comment and replaced “[…] the majority of the population is best described by B-R” by the more precise statement “[…] responses of the majority of participants are best modelled by B-R”

* p7: "the transition from unimodality to bimodality" - of what? The response distribution? The model posterior?

We clarified the statement by changing it to:
“[…] the transition of the model's posterior predictive distribution from unimodality to bimodality.”

* p7: "we use the response variance" - which variance? The variance of responses in trials in which the same stimulus was shown? Please be precise.
We thank the Reviewer for the comment. We give more specific formulation in the current version: “we used the participants' response variances at the different levels of generative noise.

* p12, "regression and inference differ" - too general use of "regression" and "inference" (in particular, as regression is a form of inference), please relativize, e.g. "Algorithmically, however, linear regression and Gaussian Process inference differ" - also for later instances of "regression" and "inference" in this paragraph.
We thank the Reviewer for the comment. Originally, we wanted to address the conceptual difference between inference and regression on a general level. Therefore, we intentionally used the general words inference and regression rather than the specific words Gaussian process models and Bayesian regression. However, we agree with the Reviewer that limiting the scope of the discussion to Gaussian processes and Bayesian regression fits the context of the paper better. We updated the paragraph in the Discussion section accordingly.

* p15, "if they have the same dominant mode, i.e. either up or down" - too colloquial and imprecise.
We agree with the Reviewer and made the wording more precise. The phrase now reads “parabolas facing either upwards or downwards”.

Additional details:

p3: "In our main experiment, we set the standard deviation of each prior model to sigma_pi = 0.1" - this is unclear, as sigma_pi hasn't been defined. Overall, this section would benefit from adding the stimulus equations (4) and (5). This would make it significantly easier to understand the generation of the stimulus. "We fixed the values of x positions ..." - do you want to say that those are the locations where the dots are shown?
We clarified the meaning of sigma_pi earlier in the text (first paragraph of the Results section) and now give pointers to Eq 4 and Eq 5.

p4, footnote 1 - shouldn't that footnote appear earlier, when you introduce the MAP-R model?
We thank the Reviewer for the comment. We believe the footnote about MAP-R and trial-by-trial noise estimation could be located after we introduce MAP-R or after we introduce the model with trial-by-trial noise estimation (B-R_sigma). We decided to keep it where it was but updated it to better fit where it is located the footnote now reads:
“Note that technically, this trial-by-trial noise estimation could also be applied to the other models such as MAP-R. However, MAP-R reduces the posterior distribution […]”

p5, "Fig 2(E) illustrates that participant's responses cover a wide range of values" - would be useful to state upfront that Fig 2(E) relates to increased sig_pi.
For clarification, we added an additional sentence: “Fig 2E illustrates the participant's responses in the condition when sigma_pi = 0.5. The participant's responses cover a wider

range of values than in the conditions of experiment 1 when sigma_pi is smaller (i.e. sigma_pi = 0.1)."

p6, Fig. 3 "Negative value favour sampling" - should be "valueS"
Corrected

p8, "because because" - repetition
Corrected

p11, "It is an interesting question whether subjects solve regression tasks with model uncertainty by taking advantage ..." - resolving model uncertainty has previously been studied under the name of "causal inference". See, for example, Koerding et al. (2007), PLoS ONE.
We thank the Reviewer for pointing out this relevant study. While it is an important step towards model inference and suggests that subjects sample from the model posterior, it leaves open the question whether subjects behave similarly in the context of function fitting. We point this out and included the reference by adding the following sentence:
"A study in the context of sensory fusion suggests the latter but it is unclear to what extend this is also the case in the domain of function fitting."

p12, "through constant feedback" - "constant feedback" makes it sound as if the feedback was the same across trials. "continuous feedback", or "feedback in every trial" might be better.
We thank the Reviewer for the comment and changed the wording to "feedback in every trial".

Supplement page 8: "... we disregarded that model" - "disregard" sounds as if you don't consider this model to be valid. Please rephrase to state that you don't consider it in the model comparison, but that it is nonetheless a valid model.
We thank the Reviewer for the comment and replaced the word "disregard" with "did not include this model in the model comparison".

Fig. 2A: please change colors to make sure that the 4 dots are visible.
We improved the visibility of the dots by changing their colour from dark gray to bright green.

Fig. 4: green line is not visible
To improve the visibility, we increased the thickness of the green line (and all other lines). Moreover, we increased the contrast between the green line, which is dark, and the colormap of the density plot by using a lighter colormap.

Reviewer #2: The authors have revised the manuscript thoroughly and addressed all of my comments. The addition of the unimodal analysis has strengthened the paper. Overall, this is a clear and thoughtful manuscript addressing an important question.

I have one concern about the new learning analyses: I am not sure it is appropriate to include all noise conditions in a single regression (Figure S3). Different amounts of learning could be expected for the different noise conditions, because there is more ambiguity for high noise, and thus observers could benefit more from learning those specific stimuli across trials. This

does seem to be the case in the data – sigma_g = 0.1 and 0.4 data points fall slightly below the unity line. I agree with the authors that the magnitude of learning is small and is unlikely to be a serious methodological concern (also considering Figure S4). Still, I would recommend performing separate statistical tests for the different noise conditions and, depending on the outcome, perhaps modify the claim in the manuscript of no learning.

We thank the Reviewer for pointing out that our analysis in Fig S3 is not sensitive the possibility that learning could occur only at some experimental conditions, e.g. the more difficult ones. To address this point, we conducted an additional analysis for each experimental condition. The results are shown in Fig S3 and Fig S4. We kept the original analysis because we believe that it is helpful to present a summarised conclusion on learning first, i.e. a conclusion based on a fit across all experimental conditions. In a second step, we provide additional analysis for each condition. We added the discussion of the results in the section "No evidence for substantial learning" in the Supplementary Information.

The additional analysis concludes that the average number of jumps between the two modes at the condition sigma_g = 0.1 is smaller in the second half of the presented repetitions than in the first half (Fig S4, (B)). Therefore, we added the phrase "except for some mild learning at \sigma_g = 0.1" in the maintext on p12.

We also simplified the labels of the axes in Fig S3 and Fig S4 and use the caption to formally explain how the shown quantities were computed.

Reviewer #3: Major point: The authors adequately respond to this point, with updated text on page 12.

Minor points: The authors have given attentive responses to all my minor points.

I have read the revised manuscript - I think that this version is significantly improved compared to the original manuscript. Overall, I think this is a rigorous study which the readers of PLoS Computational Biology will be interested in reading.
