# Supplementary material for: Bayesian regression explains how human participants handle parameter uncertainty
Source: PLoS Comput Biol. 2020 May 18;16(5):e1007886. doi: 10.1371/journal.pcbi.1007886 (PMC7259793; doi:10.1371/journal.pcbi.1007886)
Supplement: Attachment — Submitted filename: response_to_reviewers.docx [file pcbi.1007886.s003.docx]

**Editor:**

We made the following changes which are unrelated to a specific comment from the Reviewers or the Editor.

In the current version, we have reduced the number of Bayesian regression variants to two: the original B-R and B-R_sigma (which correspond to B-R4 in the old version). We believe that B-R_sigma is sufficient to argue that trial-by-trial noise estimation can potentially explain the discrepancy between the variance distribution predicted by B-R and the empirically observed variance distribution. The additional models did not bring additional insights but added to the complexity of the manuscript.

To compute the “motor noise”, sigma_m, we now use the difference between percentiles rather than the standard deviation. This method is more robust to outliers.

In the exemplary presentation of responses (Fig 2, A-D), we use only responses from participant 2. In the previous version, we used participant 2 and 3, which is less consistent than taking a single participant as example.

Major:

I have read the paper with great interest and have a few points in addition to the concerns raised by the reviewers. My main comment concerns that statement at the very end of Results that “The two additional variants of B-R perform slightly worse than the original B-R in terms of the log-likelihood”. Perhaps I misunderstood, but isn’t this in direct contradiction with the conclusion that the data support “..the idea that the generative noise is indeed jointly estimated with the quadratic parameters”? The BR_f model may provide a better fit to the variance curves (a summary statistic), but this apparently comes at the cost of not explaining other parts of the data as well as BR.

We thank the Editor for pointing out the inconsistency in our original formulation and we agree that there was an apparent contradiction between the results of the variance metric and the log likelihood metric. The reason for this apparent contradiction is that the variance plot (Fig 4) represents a summary statistics across all trials. It is not sensitive to how well a model matches individual trials. We discuss this point in the Discussion (pg. 9), please not that we renamed BR_f as B-R_sigma:

“Since in the variance analysis, we considered the response variance from all trials, the relatively better performance of B-R$_\sigma$ here did not translate into superior performance in the log likelihood analysis in which we analyzed responses on a trial-by-trial basis. B-R without noise estimation was more accurate in predicting the mean and variance of the data trial-by-trial.”

We also added a clarification in the results section (pg. 8):

“Despite the fact that B-R$_\sigma$ describes the qualitative features of the variance distribution better than B-R, it performs worse in terms of log likelihood. This shows that the low variance responses of humans and of B-R$_\sigma$ do not always coincide on a trial-by-trial basis.”

I agree with Reviewer #3 that the presentation of the model comparison results can be improved. I suggest to either go with the solution proposed by this reviewer (compute ratios w.r.t. baseline model) or go with relative values (subtract for each subject the log lh of the overall best model from the log lh off all models, such that the best model has by definition a \delta log lh of 0).  Also, i would suggest the present these results using a bar graph rather than lines (Fig. 3) and add error bars that indicate variability across subjects. Since there are only 7 subjects, you may even want to consider reporting the individual model comparison results (possibly in Supplement if it would make the main figures to cluttered).

We welcome the Editor’s remark. We changed the representation to bar plots and replaced the log likelihoods by the log likelihood with respect to B-R in (Fig 3, Fig 5, Fig 6). To better represent intersubject variability in model performance, we use the SEM across subjects and included a Bayesian random effects analysis in the Main Text (as suggested by Reviewer 1).

We also added a section on a by-subject model comparison to the Supplementary Information (Fig S1, Fig S2).

On p. 7 it is mentioned that “The data is pooled across participants”. I assume that this is only a presentation issue and that the models were still fitted to individual data sets? Please clarify. Also, *if* the models were fitted to pooled data, please justify this choice as that would be a bit strange.

We thank the Editor for the question and acknowledge that we did not make clear enough that the models did not require any fitting. To clarify this, we added a sentence on pg. 5, when we introduce the models:

“The models are fitting free because their predictions for a given stimulus $D_j$ have no free parameters.”

On pg. 11, in the Discussion, we added a more in-depth discussion of the advantages of our fitting free approach.

Finally, in the introduction it is mentioned that “Most […] experimental studies can be cast into the problem of estimating a hidden quantity from sensory input”. It may be worth noting that we have found evidence for Bayesian inference in various perceptual tasks that go beyond simple estimation, including visual search (e.g. Ma et al. 2011, Nature Neuroscience; Stendgård & Van den Berg 2019), same/different discrimination (Van den Berg et al 2012, PNAS), and change detection (Keshvari et al 2012, PLoS One).

We are grateful for these helpful references and changed the sentence to “Some experimental studies have considered complex tasks such as Ma et al. 2011, Nature Neuroscience; Stendgård & Van den Berg 2019), same-different discrimination (Van den Berg et al 2012, PNAS), and change detection (Keshvari et al 2012, PLoS One) but most […]”

Minor:

I think that the abbreviation “DNNs” on p. 2 was never introduced

We replaced “DNNs” by “Deep Neuronal Networks (DNNs)”

Sigma_m is in the supplement referred to as “motor noise”, but in the main text it is explained that it more broadly captures “internal sources of noise arising during neural processing, decision making and the execution of motor action”. I prefer the broader interpretation of sigma_m and believe it would be useful to add a reference to Drugowitsch 2016 (Neuron) and Beck, Ma, et al 2012 (Neuron) here.

We thank the Editor for the helpful remark and references. We believe it would be a good compromise between brevity and precision to define what we mean by motor noise very clearly but stick to the short and simple name “motor noise”. In particular, we believe that the term motor noise conveys clearly that we model it as a participant specific source of noise and that it is not related to any specific model. We hope our introduction of the motor noise on pg. 5 in the current version is satisfactory:

“To model participants' responses, we also included the internal sources of noise arising during neural processing, decision making and the execution of motor action \cite{beck2012not,drugowitsch2016computational} collectively called motor noise for brevity.”

**Reviewer #1:** The authors ask if human participants feature behaviour that is better explained by Bayesian regression rather than simpler alternatives/heuristics. The specific feature of Bayesian regression is that predictions need to take into account the uncertainty in the inferred regression coefficients. The authors test this by providing subjects with noisy point-wise observations of a quadratic function, and ask for the participants' best guess when extrapolating the (unobserved) function to more extreme values. They demonstrate that the participants' extrapolations are best explained by a Bayesian regression model rather than alternative models, such as maximum likelihood or maximum a-posteriori. This is, to my knowledge, the first demonstration that humans are able to take account of their uncertainty in model parameters when making predictions. Thus, it is novel, original, and should be of interest to a wide audience.

Unfortunately, the current manuscript leaves multiple, potentially confounding questions unanswered:

First, the authors seem to implicitly assume that predictions are made by drawing samples from the posterior predictive density (after which some motor noise is added). This is just one possible way of turning posteriors into decisions. Bayesian decision theory posits that decisions ought to be made by picking an estimate that minimizes a particular expected loss under the posterior. The squared loss, for example, would result in deciding according to the posterior mean (see Bishop (2016) and Kording & Wolpert (2004)). There exists no loss function, however, that makes posterior sampling the optimal strategy. Therefore, Bayesian decision theory does not currently justify the performed analysis. One could suggest that the participants do not act according to Bayesian decision theory, and instead sample from this posterior. However, this does not conform to recent work (Drugowitsch et al. (2016)) showing that behavioral variability seems to arise from noisy inference rather than noisy decisions. Thus, I urge the authors to elaborate in more detail how they believe posteriors are turned into choices, and potentially investigate if noise in the inference itself could result in the behavior that they aim to describe.

We are very grateful for the Reviewer’s stimulating remark about Bayesian decision theory and noisy inference. Based on it, we substantially updated the manuscript by including two different decision models, i.e., probability matching and Bayesian decision theory.

In the Introduction (pg 2), we added a paragraph that mentions previous work on Bayesian decision theory (including noisy inference) and probability matching.

We introduce both decision models (loss based and probability matching) in a dedicated subsection (pg. 5).

We ran an additional model comparison (Fig 3C) and concluded that loss-based models perform worse than probability matching. Constrained on unimodal responses, the loss-based models performed better but B-R with sampling was still the best model summed over all conditions and participants.

We included a section in the Supplementary Information in which we apply the noisy inference framework of Drugowitsch et al (2016) in the context of our task. Indeed, noisy inference can explain the transition to the bimodal response distribution as a function of the generative noise. The computation of the mixing coefficients in noisy inference is tightly linked to the mixing coefficient in Bayesian regression. We added a paragraph to the Discussion (pg. 11) to highlight this link. However, we did not test noisy inference quantitatively because we wanted to avoid the additional complexity of introducing models with free parameters.

In an additional Discussion paragraph (pg. 11) we suggest that a sample based representation of the posterior predictive distribution could also reconcile Bayesian decision theory with the bimodal response distribution of the participants.

Second, the model comparison results seem to be driven by the bimodality of participants' responses (i.e., convex vs. concave function), which appears only supported by Bayesian regression. That by itself would be an interesting result, but needs further investigation.

If you would restrict responses to the single, dominant mode (e.g., ignoring all trials in which the other mode was chosen), would the Bayesian regression model still win the model comparison?

Would a simpler model that only considers two possible functions (i.e., convex or concave?) + some motor noise perform worse than the Bayesian model?

We thank the Reviewer for pointing out the importance of investigating the unimodal case. We followed the Reviewer’s suggestion and conducted an additional model comparison on a unimodally constrained data set. We show the results in a new figure (Fig 6) and discuss the results in a dedicated subsection of the Results section. Depending on the experimental conditions, the unimodal responses were either best described by B-R; or all models (including the ones that used loss-based decision making) performed similarly well. Summed over participants and conditions, B-R with sampling was the best model. Thus, we concluded that B-R was overall supported by the unimodal analysis.

On the unimodal data set, the model suggested by the Reviewer corresponded to L:P-R, i.e. prior regression with a loss-based decision model. L:P-R did generally not perform better than B-R (Fig 6B and Fig S2).

Third, model comparison is performed on the mean log-likelihoods. Even though Fig. 3 might suggest otherwise, this doesn't per se exclude the possibility that the model comparison isn't driven by a few participants. I would suggest two modifications.

First, the models should be compared in terms of log-likelihood ratios, using the preferred model as baseline. Then, all measures are relative to the model fit of this preferred model, and negative values indicate a worse model fit of alternative models. Averaging across those would correspond to a more informative within-subject comparison. Second, to exclude the possibility of few participants driving the model comparison I would suggest additionally performing a Bayesian random effects model comparison (see Stephan, Penny et al. (2009)).

We thank the Reviewer for the helpful comment. As suggested, we changed the performance measure of the models to the log likelihood with respect to B-R (Fig 3, Fig 5, Fig 6 and Fig S1, Fig S2, Fig S4) throughout the manuscript. We also added the Bayesian random effects analysis in Fig 3B and Fig 6. In the Supplementary Information, we included a subject-level analysis Fig S1 and Fig S2.

The Bayesian random effects analysis was particularly helpful to discover intersubject variability in the case of MAP-R at the low noise condition (Fig 3B).

Minor comments:

Abstract: "The quadratic parameter was drawn from a prior distribution, unknown to the observer" - what was unknown to the observer? The quadratic parameter? Or that this parameter was drawn from a prior distribution?

Indeed, we agree with the Reviewer that the formulation was not clear. The current version of the abstract does not mention what is unknown to the subjects explicitly. This removes the potential source of confusion. The new sentence reads: “The quadratic parameter was drawn from a bimodal prior distribution.”

p3, A novel paradigm to test regression: I found this section generally confusing, and I encourage the authors to revise the presentation order to make stimuli and experiment clearer. I understand that you have 20 4-dot stimuli with the same underlying wj. For a fixed noise level, did they also have the exact same noise instantiation - i.e., was the stimulus the same on the screen? Were they the same across participants? Furthermore, you repeat each stimulus 20 times, which would imply 400 stimuli overall. How were these 400 stimuli distributed across the different blocks of different noise level? What about the 10 practice trials?

We welcome the comment and rewrote the section (pg. 3) in order to make it more accessible.

p4, Fig 2 caption: "Contours indicate the equiprobable responses predicted by ..." - not precise as responses only happen at x=2. Is this the predicted response distribution if subjects were asked to respond at different x's?

For (D), are those the response distributions corresponding to the dot pattern in (A)?

We thank the Reviewer for pointing out that we were not clear enough. Indeed, (A) and (D) are derived from the same dot pattern. To address the comment, we updated the caption of Fig 2:

“A sample stimulus (black dots) at high noise level (\sigma_g = 0.4). For this specific stimulus, contours indicate the response distributions predicted by ML-R, MAP-R, B-R and B-R_\sigma (not shown to the participant) at various x^\star. At x^\star = 2, we recorded the participant's responses (gray dots). The cross section at x^\star=2 is shown in D.”

p5, "To complement the log-likelihood analysis [...], we used the variance" - which variance?

To clarify, we added “variance of the responses.”

p6, Fig. 4 caption: "The errors of the other models are too small to show" - errors? Or variance? SEM? SD?

We now indicate that errors are computed as SEM in Fig 3, Fig 6 and in Fig S1, Fig S2.

p9, "In each trial, the participants carried out [...]" - you only know that their behavior was compatible with the proposed computations, but not the exact computations participants performed to feature this behavior.

We agree with the reviewer on this point and adopted a more careful wording: “In each trial, we model the participant's computation by a consecutive inference and prediction step. During the inference step, the model assumes that the participant infers information about the quadratic parameter …”

p9, "in Bayesian regression generalizes Eq (8" - ")" missing

The sentence is no longer present in the current version.

p9, "as in ordinary B-R (Eq (8)" - ")" missing

The sentence is no longer present in the current version.

p10, "(the log of Eq (10)" - ")" missing

We added “)”

Fig 4 B/C/D: please consider horizontal jitter of the grey dots instead of blurring them.

We thank the Reviewer for the good idea and added jitter.

SI, general: many of the derivations are standard in the Bayesian literature, and their details can be skipped or they can be simplified (e.g., Bishop (2016), Appendix B, "Gaussian")

We removed the general derivation from the appendix and replaced it by the equations used to generate the predictive distributions.

SI, p2: "see ??" - broken ref

The sentence is no longer present in the current version.

SI, p3, "Fig 2(A) / Fig 2(B)" - should this refer to Fig. S2?

The sentence is no longer present in the current version.

SI, p4, "[...] because our mixed Gaussian prior is the conjugate prior of the Gaussian likelihood" - "is A conjugate prior", as there are multiple.

The sentence is no longer present in the current version.

SI, p6, "[...], we the posterior maintains" - the "we" should be removed

The sentence is no longer present in the current version.

SI, p8, Derivation of full Bayesian regression: wouldn't a normal inverse-gamma mixture provide a conjugate prior for this case? This would lead to analytical posteriors.

We agree with the Reviewer that our choice was not obvious and would have needed a better motivation. In the current version, full B-R was removed. However, to explain our original rationale: we opted against the inverse gamma prior to stay fitting free. In an exponential prior, the only free parameter can be set via the mean which we assumed to match the true value of the hyperparameter.

**Reviewer #2:** The authors asked how humans handle parameter uncertainty in a regression task. Observers had to extrapolate from 4 points generated from a noisy quadratic function to predict the y-coordinate of a 5th point whose x-coordinate was given. They compared Bayesian regression to prior regression, ML regression, and MAP regression, and found the best performance for Bayesian regression models fit to the human data. The question is important, as function estimation is a basic and general task. The paper was very clearly written and technically proficient. The evaluation of the models by two separate methods (log likelihood and response variance) was a strength. All methods details were provided. However, I wondered whether the claim that humans use Bayesian regression depended critically on the specifics of the task and noise assumptions. I also wondered how this paper goes beyond previous arguments that humans use Bayesian regression.

Major comments:

1. Novelty: Lucas et al. 2015, Psychonomic Bulletin & Review, argue that previous models of how humans do function estimation can be recast as Bayesian regression. They also compare performance of various models and show that Gaussian process models, which are closely related to Bayesian regression models, fit human data well. How does the current work go beyond these previous arguments for Bayesian regression?

We thank the Reviewer for pointing out that the study of Lucas et al. 2015 is closely related to our work. On pg. 11, in the Discussion, we included a paragraph to highlight why our rationale of simplicity is, in our view, better suited for studying parameter uncertainty:

“For a given generative model, Bayesian regression and other regression models prescribe how to make prediction when parameter uncertainty is present. For example, MAP-R uses a point estimate of the posterior while B-R uses the entire posterior. Thus, the performance of regression models in a model comparison depends on two factors, the ability of the regression model to describe how humans handle uncertainty and the degree to which the underlying generative model is able to capture the data. The predictions of the regression models in our study are limited in that they assume a parabolic generative model. A previous study on Bayesian regression reiterated its formal equivalence to Gaussian processes and demonstrated its flexibility in modelling human responses in a complex function fitting task \cite{lucas2015rational}. In the case of the Bayesian regression model, the authors fit various hyperparameters and it is unclear how they controlled for the complexity of the fit. Thus, the study could not answer if the Bayesian regression model performed well because of its flexibility in representing different generative models or because it captured how humans process parameter uncertainty. In our work, we removed the confounding factor by enforcing a simple generative model through constant feedback. Instead we remained fitting free and could, thus, study directly how participants processed parameter uncertainty.”

2. It seems that the MAP model failed primarily because it did not capture the bimodal distribution of observers’ responses. In this task, the parameter of interest, the coefficient on the quadratic term of the function, was drawn from a bimodal prior distribution, so that the parabola sometimes opened upward and sometimes opened downward. For the same 4-dot stimulus, human observers sometimes extrapolated for a downward parabola and sometimes for an upward parabola, whereas the MAP model always extrapolated in just one direction. But if I understood correctly, all models knew the generative noise, unlike the human observers. How much do the unimodal predictions of the MAP (and ML) models rely on this assumption? That is, if the noise were estimated trial by trial, would this stochasticity lead to bimodal predictions even for MAP and ML models? This concern seems especially relevant given that the authors show better performance for versions of the Bayesian model that estimate the generative noise trial by trial, and it seems that this possibility was not tested for the non-Bayesian models.

We thank the Reviewer for raising the interesting question if MAP-R (and ML-R) could produce bimodal responses via a noise estimation mechanism. To explain why this is not possible, we’d like to highlight that the computation of the model predictions involves no stochasticity. For each unique stimulus, we compute the prediction of each model only once. To turn any of the models into a model with noise estimation, we first estimate the generative noise from the stimulus and then run the model with the estimated noise rather the true hyper parameter. This is how we derive B-R with noise estimation (B-R_sigma).

The Reviewer raised the question if ML-R or MAP-R with noise estimation could produce a bimodal distribution. This is not possible because these models produce unimodal distributions for any value of the noise and, for each stimulus, the noise estimation variants use a (deterministically) determined point estimate of the noise.

To clarify this point, we adapted the paragraph in which we introduce the models (pg. 4):

“Relaxing the assumption that the true generative noise is known, we included an additional variant of B-R that (deterministically) estimates the generative noise $\hat{\sigma}_g$ from the current stimulus $D_j$. We indicate this variant of B-R with a subscript: B-R$_\sigma$. Since MAP-R reduces the posterior to a point estimate, by-trial noise estimation does not change its prediction substantially, i.e. it only shifts the unimodal prediction between ML-R at low noise estimates and the close by prior mode at high noise estimates. Thus, we did not consider it further.”

As an additional remark (not mentioned in the current version): Even if the noise estimation involved stochasticity such that the estimated value was different upon every presentation of the unique stimulus ML-R and MAP-R could not produce bimodal responses. The reason is that the sign of the predicted parabola is independent of the noise level. The ML estimate of the parameter w is independent of the noise level. In MAP-R, the generative noise balances the prior of w with the ML-prediction of w. Since the prior is symmetrical any non-zero noise estimate yields a MAP parabola that has the same sign as the ML parabola. The value of the noise only determines how close the final value is to either one of the prior modes or the ML value.

3. Relatedly, is there any aspect of the data that argues for Bayesian regression that is not dependent on the bimodal pattern of extrapolation reports? It seems that both the log likelihood results and the variance results were driven by the bimodal pattern, which raises the question of whether the result would generalize to a case where the prior is unimodal. This seems important, as unimodal priors on parameters are probably more typical in natural tasks.

We thank the reviewer for the important comment. Indeed, the transition from unimodal to bimodal is the central signature of B-R in our setup. We’d like to point out that bimodality is not atypical but a salient feature in human responses (e.g. Kalish et al. 2004). To the question if B-R described unimodal responses as well, we added two types of analysis to the manuscript.

First, to remove the effect of bimodality on model performance we conditioned the dataset to unimodal responses. On the conditioned dataset, we repeated the model comparison. The results are reported in a new results section, in particular Fig 6. Across all participants and conditions, B-R emerged as the winning model. However, at the medium (0.1) and high noise (0.4) condition, all models showed rather similar performance and B-R was not superior. The results also differed on a by-subject level (see Supplementary Information).

Secondly, we discuss a decomposition of the response variance into a unimodal and a bimodal contribution (Fig 5). While the bimodal contribution of the variance in the data matched the predictions of B-R, the unimodal variance tends to be lower than what B-R predicts at high noise conditions (Fig 5C).

In conclusion, the evidence for B-R on the unimodal data is weaker but the model comparison still favoured B-R over all.

4. The experiment consisted of 20 repetitions of each of 20 4-dot stimuli. Feedback was presented after every trial, which created the possibility that the observers could learn a direct mapping between a certain dot configuration and an extrapolated point over the course of the experiment. I wondered whether such learning could create the appearance of Bayesian performance, and more generally whether there was any evidence for learning over the course of the experiment.

We are grateful to the Reviewer for bringing up this very important point and think that the current version has benefited a lot from addressing. To address the question of learning, we conducted an additional analysis (Supplementary Information) and included two additional Figures (Fig S3 and Fig S4).

To address the question if participants memorised the generative parabola of a given stimulus over time, we considered the distance of the response to the generative parabola as a function of repetitions. We did not find substantial evidence for learning (Fig S3 (A)). A similar analysis showed that subjects oscillate between both modes even during later repetitions of the same stimulus (Fig S3 (B)). We considered the log likelihood performance over time in Fig S4 and found no clear indication of learning.

Therefore, we conclude in the Discussion (pg 11): “Indeed, after having run the experiments, we found that there was no substantial learning taking place between the first and the last trials (see Supplementary Information)”

5. The B-R3 model seemed a bit ad-hoc. Is there a theoretical reason for estimating the noise based on two sets of 3 points rather than all 4 points (the B-R4 model reported in the supplement, which did not perform as well)?

We agree with the Reviewer’s observation. In fact, the model was motivated by a conversation with one of the participants. In the current version, we dropped B-R3 and full B-R because they make essentially the same point as B-R4: by-trial noise estimation is one possibility to explain the discrepancy in the observed and predicted response variance. In the current version, we instead analyse the different contributions to the B-R variance more in detail (see new Fig 5).

Minor comments:

1. The significance statement is quite vague. It seems like it could apply to almost any Bayesian modeling paper.

We agree with the Reviewer and rewrote it with the aim of making it more specific.

2. To motivate the study, it may be worth pointing out in the intro that while “many aspects of cognition are indeed well described by Bayesian statistics” (p. 2), many are not (Rahnev & Denison 2018).

We are thankful that Reviewer pointed out these relevant studies and included them (pg. 2).

3. “This feedback enabled the participants to learn both the prior and the generative model.” (p. 3) What is the evidence that these were learned successfully?

We welcome the Reviewer’s question. Indeed, there was only indirect evidence of learning. We added a sentence on pg 5, after discussing the example trials: “The observation that at $\sigma_g = 0.03$ MAP-R matched the participants’ responses often with high accuracy provided implicit evidence that participants used the prior and had learned parabolic generative model.”

4. It may be helpful to explain why the MAP model does not predict a bimodal distribution even though it uses the prior.

We agree with the Reviewer and added a clarifying sentence when introducing MAP-R (pg. 4): “Despite using the bimodal prior it cannot produce bimodal responses because it relies on a point estimate of $w$.”

5. For the variance analysis, why was the variance within a given stimulus (observers) compared to the variance generated across a large number of stimuli (models), rather than comparing variance from the same source?

We welcome the Reviewer’s comment and hope we could make our rationale clear to the reader in the current version: We decided to generate a large number of stimuli for many values of the generative noise sigma_g to show how the variance distribution evolves as a function of this parameter. Particularly the difference between the variance distribution of B-R and B-R$_\sigma$ in Fig 4 would not become as obvious if we had only used the stimuli of the experiments. We added an explanation for this choice when we introduce the Fig 4 (pg 7):

”To achieve a higher resolution and show the dynamic of the variance as function of the noise, we generated 5000 unique stimuli from densely spaced $\sigma_g$ instead of relying on the small number of stimuli and noise levels used in the experiment”

6. For B-R3, how was the ML estimate of the generative noise obtained? (p. 9) Does the procedure assume that w is known?

We agree with Reviewer that this is a potential source of confusion. To address this, we moved the exact mechanism from the Supplementary Information to the Methods (pg 13, Eq 10). When we introduce B-R with noise estimation (pg 4), we now explicitly mention that only the stimulus is used for the noise estimation:

“Relaxing this assumption, we included an additional variant of B-R that uses only the stimulus of the current trial to estimate the generative noise $\hat{\sigma}_g$.”

7. Some figure references were missing or in a different format (e.g. “Bottom right” vs. “D”).

We thank the Reviewer for pointing out this inconsistency and corrected it.

**Reviewer #3**: This is a very neat paper which made me wish I’d thought of doing that experiment. Essentially, the manuscript shows some reasonably convincing evidence that people can complete a simple regression problem in a Bayesian manner.

Major points

I felt that the manuscript treated the division between the inferential tasks of model comparison, regression, prediction, parameter estimation, posterior prediction, as too sharply divided. While these _concepts_ are distinct, once we start thinking about people completing real behavioural tasks, then I think it becomes a bit less clear to claim the completion of a task only involves one inferential concept and not others. I think the authors could make their claims more robust by adding more reasoning why this task should be considered as regression as opposed to prediction. Someone could make a reasonable case that the participants are doing posterior prediction here, so the case for regression could be firmed up somewhat. Someone may argue that regression is not a 'core' inferential task in that there is just model learning, parameter estimation, and posterior prediction. If so, then the paper demonstrates that people can learn a parabolic model and do parameter estimation and/or posterior prediction. I think the authors could perhaps update the manuscript to make their claims more robust to someone with these views.

We welcome the Reviewer’s remark and agree that Bayesian regression and posterior prediction represent two equivalent formulations of the same computational goal. However, we think that they represent to different algorithmic paradigms and that it is thus useful to keep them conceptually separated. Regression reduces training data to a set parameters while inference treats the training data as observed variable (used in an inversion in Bayes’ theorem). This is particularly important when memory constraints play a role. For example, when training data is shown sequentially the regression framework can update its posterior without increasing the memory demand. In the inference perspective, the memory demand increases linearly with time. We make this argument more in detail in a new paragraph in the Discussion (pg 12) and hope that it provides fruitful inputs for how these two perspectives could be disentangled experimentally.

Minor points

The model comparison is nice, but I think it would also be useful very early on to include a short intuitive statement about what kind of results would really support or disconfirm that people are doing this regression task in a Bayesian manner.

We thank the Reviewer for this helpful comment. In the current version, we included the following sentence at the end of the Introduction (pg 3) “The rationale is that the higher the noise level, the higher the uncertainty about the correct parameter and, according to Bayesian regression, the more participants should rely on the prior and produce a bimodal response distribution.”

Clarify if participants were naive to the goals of the study.

We added the word “naïve” in the Methods.

This is touched upon in the manuscript, but it might be worth elaborating or speculating a little more about what might happen outside of a toy scenario where the participants know the data generating model. Presumably people would have to simultaneously conduct parameter estimation (of multiple models) and do inference over models.

We appreciate the Reviewer’s suggestion to speculate about the implications of our study for real world regression tasks. To address this point, we included two additional paragraphs to the Discussion section.

The first paragraph (pg. 11) we discuss how to naturally extend the B-R framework to the case of model uncertainty.

In the second paragraph (pg. 11), we comment on the challenge of high dimensional parameter spaces for Bayesian regression. We suggest that a sampling based representation of probability distributions would support efficient scaling in high dimensions and link to recent findings in the computational neuroscience literature on neuronal sampling.

Unless I've misunderstood, a core part of this manuscript is the bimodality of the parameter distribution. I felt that this was not given much attention in the manuscript so perhaps there is some scope for clarification.

We are grateful for pointing this out and agree. In the current version, we mention the bimodal prior already in the abstract and made it a more prominent point of reference in the current version, as the following examples illustrate:

The sentence about the transition to a bimodal distribution (see answer to minor comment 1) at the end of the Introduction (pg 3) sets the stage for bimodality as a signature of B-R in our study.

When we introduce the models, we now comment on their capability of producing bimodal answers, e.g. in the case of MAP-R (pg. 4): “Despite using the bimodal prior it cannot produce bimodal responses because it relies on a point estimate of $w$.”

On pg 6, when we explain the results in Fig 3A, we now comment on bimodality very clearly:

“As the task difficulty increases (left to right), P-R performance approaches that of B-R. This is because the parameter uncertainty encoded in the prior becomes more important and the response distribution becomes more bimodal. Neither MAP-R nor ML-R can capture this and therefore perform poorly.”

When we motivate the variance analysis (pg 7), we mention the expectation of a transition to bimodality and its importance in our setup: “A key characteristic of B-R is the transition from unimodality to bimodality as $\sigma_g$ increases, i.e., as the data become less informative. To analyse this transition, we used the response variance.”

In the first paragraph of the Discussion (pg 10), we summarise our key insights and highlight the role of bimodality again: “An analysis of the response variance showed that the model comparison was mainly driven by the

transition from unimodal to bimodal responses. Only the B-R variants were able to capture this aspect of the data.”

Along the lines of the previous point, I was a bit unsure how to interpret the density plot for B-R in figure 2-A. The methods spell out that the mixture of up and downwards parabolas was 50%. This is not the case in this density plot, so presumably this plot is the best fit to a given participant? Perhaps this point of confusion on my part can help the authors with clarifications in the main text and/or figure legend.

We thank the Reviewer for the constructive feedback and agree that the caption text was not sufficiently clear.

To address this, we now point out more clearly in the caption of Fig 2 that the response distribution is the posterior predictive at different x-positions and that they are derived from a single stimulus (rather than encoding the prior):

“A sample stimulus (black dots) at high noise level ($\sigma_g = 0.4$). For this specific stimulus, contours indicate the response distributions predicted by ML-R, MAP-R, B-R and B-R$_\sigma$ (not shown to the participant) at various $x^\star$. At $x^\star = 2$, we recorded the participant's responses (gray dots). The cross section at $x^\star=2$ is shown in D.”

The Reviewer proposed that we were showing the “best fit to a given participant.” We’d like to point out that our study did not involve the fitting of hyperparameters to the response data. To clarify this, we added a sentence on pg. 5, when we introduce the models:

“The models are fitting free because their predictions for a given stimulus $D_j$ have no free parameters.”

On pg. 11, in the Discussion, we added a more in-depth discussion of the advantages of our fitting free approach.

Possibly a pedantic point, but Panels B-D in Figure D could feasibly be rotated so the y coordinate is on the y axis, to make it a little more intuitive. But this is just a suggestion for consideration.

We thank the Reviewer for the this thoughtful suggestion. However, we decided to keep the axis as before because to us they felt like the more natural way to visualise distributions for us.
